# Solitary Lymphatic Metastatic Lesion From Dorsal Hand Cutaneous Squamous Cell Carcinoma: A Diagnostic Dilemma Between In-Transit and Interval Nodal Metastasis

**DOI:** 10.7759/cureus.103826

**Published:** 2026-02-18

**Authors:** Yuto Yamamura, Kazuyasu Fujii, Kazutoshi Nishimura, Chisa Nakashima, Shunya Usui, Atsushi Otsuka

**Affiliations:** 1 Dermatology, Kindai University Hospital, Osaka, JPN

**Keywords:** cutaneous squamous cell carcinoma, ectopic nodal metastasis, immunocompetent patient, in-transit metastasis, upper limb

## Abstract

Lymphatic metastatic lesions arising between a primary cutaneous squamous cell carcinoma (cSCC) and the regional nodal basin present a diagnostic dilemma between in-transit metastasis (ITM) and interval nodal metastasis. We report the case of an immunocompetent octogenarian woman with a well-differentiated cSCC on the dorsum of the hand that was excised with clear margins. Six months later, a solitary subcutaneous nodule developed along the ipsilateral upper arm. Magnetic resonance imaging suggested a benign soft-tissue tumor, and an excisional biopsy was performed. Histopathological examination revealed a moderately differentiated squamous cell carcinoma morphologically similar to the primary lesion, located within a lymph node-like structure containing residual lymphoid architecture. Positron emission tomography-computed tomography showed no additional nodal or distant metastases. Based on the anatomical location along the lymphatic drainage pathway and clinicopathological findings, the lesion was considered a lymphatic metastatic deposit with features overlapping ITM and interval nodal metastasis. The patient received adjuvant radiotherapy and remains disease-free at six months of follow-up. This case highlights the diagnostic challenge in distinguishing ITM from interval nodal metastasis in cSCC and underscores the importance of careful clinicopathological correlation when evaluating solitary subcutaneous lesions arising along lymphatic drainage pathways.

## Introduction

In-transit metastasis (ITM) is a form of lymphatic dissemination characterized by dermal or subcutaneous tumor deposits that develop between the primary lesion and the regional lymph nodes [1]. It is thought to result from the proliferation of tumor cells migrating along lymphatic channels and is recognized as a potential precursor of local recurrence or distant metastasis [2].

In malignant melanoma, ITM occurs relatively frequently and has well-established prognostic and therapeutic significance. The 8th edition of the American Joint Committee on Cancer (AJCC) staging system classifies ITM, together with satellite and microsatellite lesions, as an independent N category, forming a key element of treatment planning [3,4].

In contrast, ITM in cutaneous squamous cell carcinoma (cSCC) is exceedingly uncommon and is not defined as a separate entity in the current AJCC classification or major clinical guidelines [5]. Most reported cases originate from the head and neck region, often in immunocompromised patients, and carry a prognosis similar to that of node-positive disease [5-7]. Thus, although rare, ITM represents a clinically meaningful pattern of metastasis in cSCC.

Herein, we describe a rare case of well-differentiated cSCC of the dorsal forearm that developed a solitary lymphatic metastatic lesion in the subcutaneous tissue of the ipsilateral upper arm in an immunocompetent patient, clinically consistent with lymphatic metastasis along the drainage pathway. Histopathological evaluation revealed residual lymphoid architecture within the lesion, highlighting the diagnostic challenge in distinguishing ITM from interval or ectopic nodal metastasis.

## Case presentation

**Figure 1:**
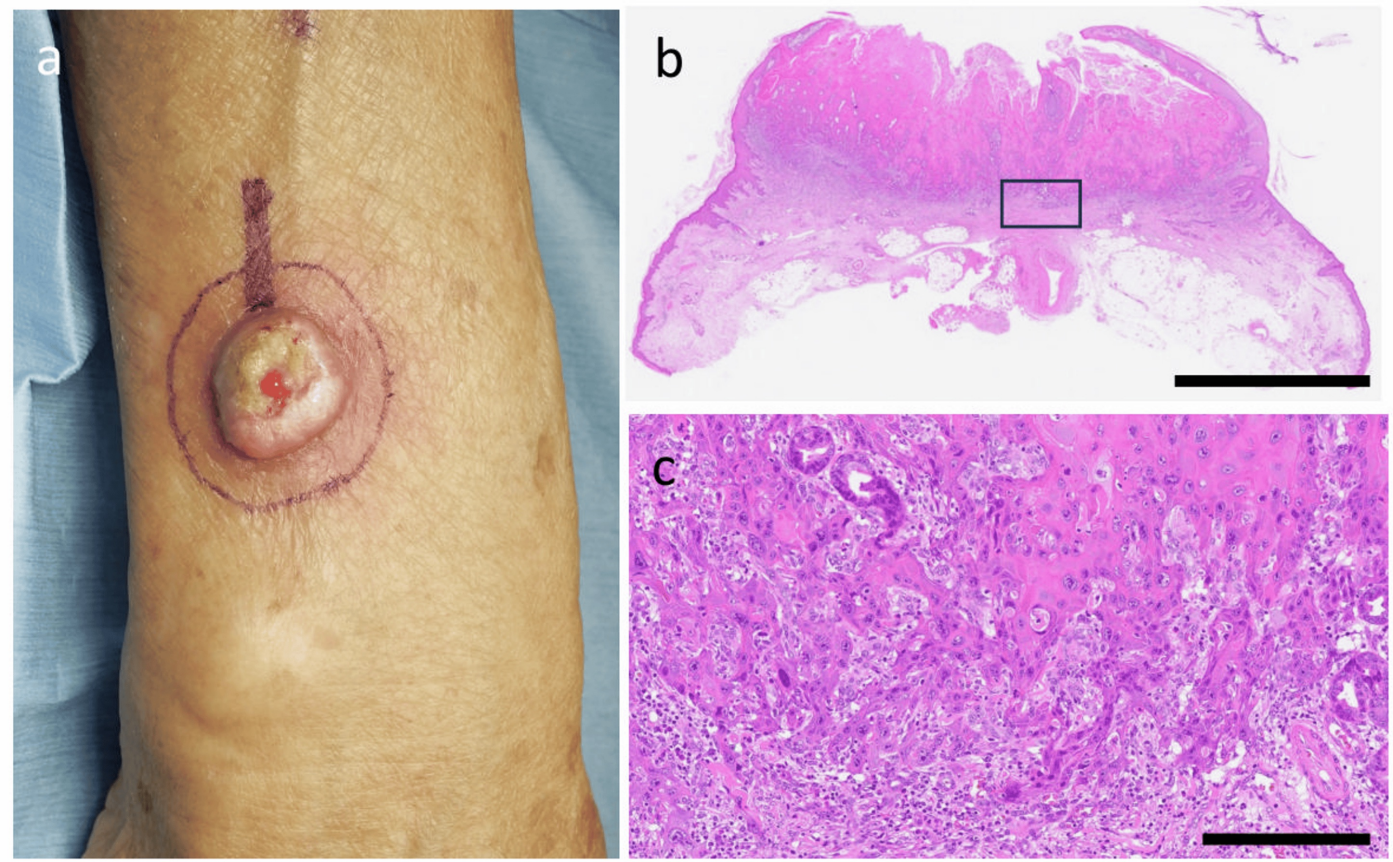
Clinical and histopathological findings of the primary lesion. (a) A keratotic nodule with a central ulcer on the dorsum of the right forearm. The planned 5 mm surgical margin was marked before excision. (b) Low-power view showing a crateriform tumor with an exo-endophytic growth pattern, consistent with keratoacanthoma-like squamous cell carcinoma (H&E, scale bar = 5 mm). The boxed area corresponds to (c). (c) High-power view of the boxed area in (b), showing well-differentiated atypical squamous cells with keratinization and intercellular bridges (H&E, scale bar = 250 µm).

An immunocompetent octogenarian woman presented with a reddish papule on the dorsum of the right forearm. She was referred to our department with a clinical suspicion of cSCC. A partial biopsy confirmed the diagnosis of cSCC, and the lesion was excised under local anesthesia with a 5 mm surgical margin (Figure 1a). Histopathological examination revealed a well-differentiated squamous cell carcinoma with a tumor thickness of 3.5 mm. No evidence of perineural or lymphovascular invasion was observed, and all surgical margins were negative (Figure 1b, 1c).

**Figure 2:**
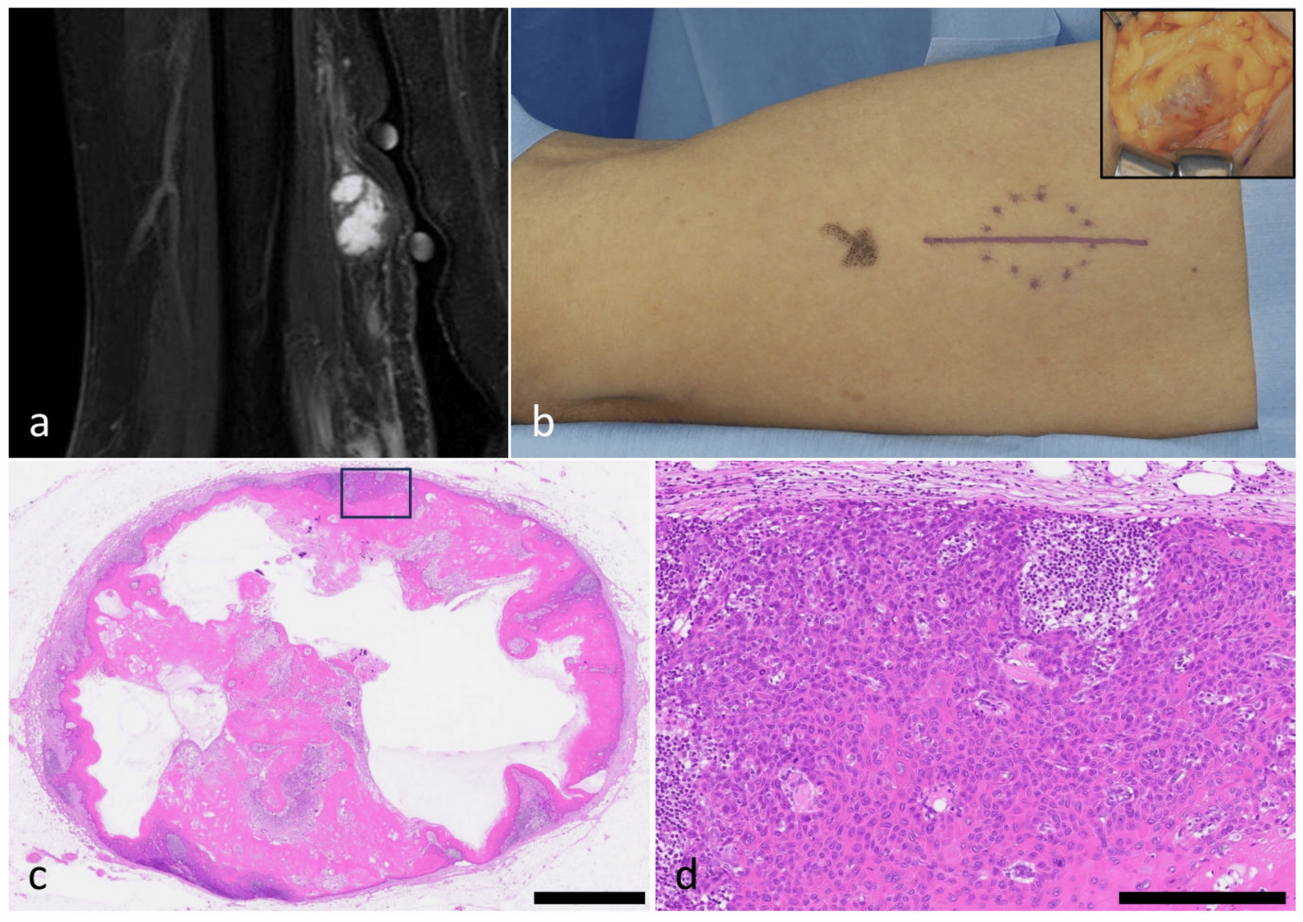
Clinical, radiological, and histopathological findings of the metastatic lesion. (a) MRI (STIR) shows a well-circumscribed, smooth subcutaneous nodule (~15 mm) in the medial upper arm. The lesion demonstrates high signal intensity on STIR with internal septations and mildly high signal on T1-weighted images, findings suggestive of a benign nerve sheath tumor or a myxoid soft-tissue neoplasm. (b) Subcutaneous nodule on the right upper arm (inset: intraoperative view of the resected nodule). (c) Low-power view showing a well-circumscribed dermal and subcutaneous tumor nodule with partial preservation of lymph node-like architecture (H&E, scale bar = 2.5 mm). The boxed area corresponds to (d). (d) High-power view of the boxed area in (c), showing moderately differentiated squamous cell carcinoma with keratinization and intercellular bridges. Residual lymphoid-like structures are also observed adjacent to the tumor nests (H&E, scale bar = 250 µm). MRI, magnetic resonance imaging; STIR, short tau inversion recovery.

Six months after surgery, a painless subcutaneous nodule developed on the medial aspect of the right upper arm. Magnetic resonance imaging (MRI) showed a well-circumscribed lesion with high signal intensity on short tau inversion recovery (STIR) and mildly high signal on T1-weighted images, suggesting a benign nerve sheath tumor or a myxoid soft-tissue neoplasm (Figure 2a). Because a definitive diagnosis could not be established, an excisional biopsy was performed (Figure 2b).

Histopathology revealed a moderately differentiated squamous cell carcinoma morphologically similar to the primary lesion (Figure 2c, 2d). The tumor was located within a lymph node-like structure; however, it was difficult to determine whether the lesion represented a lymphatic or nodal metastasis.

Positron emission tomography-computed tomography demonstrated no evidence of additional lymph node or distant metastases, nor any findings suggestive of other malignancies. Based on these clinical and anatomical findings, the lesion was considered clinically consistent with lymphatic metastasis, most compatible with lymphatic metastasis along the drainage pathway. The patient subsequently received adjuvant radiotherapy with a total dose of 60 Gy in 30 fractions, and she remains disease-free six months after treatment.

## Discussion

Metastatic lesions arising along lymphatic drainage pathways from cSCC pose a diagnostic dilemma between ITM and interval nodal metastasis. While previous reports have predominantly described head and neck primaries in immunosuppressed individuals [1,5,7,8], occurrences in immunocompetent patients with limb lesions are exceedingly uncommon. However, metastatic lesions developing along lymphatic drainage pathways may also represent interval or ectopic nodal metastasis, and distinguishing these entities from true ITM can be challenging in clinical practice. In previous series, larger (≥20 mm) or multiple ITMs have been associated with poorer prognosis, suggesting that the number and size of lesions may influence clinical outcomes [6,8].

This case is unique in two key aspects. First, it occurred in an immunocompetent elderly woman without a history of chronic disease or immunosuppressive therapy - conditions typically associated with ITM development. Second, the metastatic lesion appeared as a solitary, well-circumscribed subcutaneous nodule in the upper arm, rather than as multiple or diffuse lesions. Given the short follow-up period, no conclusions regarding the biological behavior or prognosis can be drawn. Complete surgical excision remains the cornerstone of localized lymphatic metastatic disease, while systemic immunotherapy is reserved for unresectable or recurrent cases [9].

Histologically, the metastatic focus was situated within a lymph node-like structure containing residual follicular elements, blurring the boundary between ITM and ectopic nodal metastasis. This morphological overlap underscores a key diagnostic challenge: the precise distinction between these two entities remains ambiguous, even in contemporary literature [1,5-7] where ITM has been variably defined as a dermal or subcutaneous tumor deposit occurring between the primary tumor and the regional nodal basin. In such situations, clinicopathological correlation, including anatomical location along the lymphatic drainage route and the presence of residual lymphoid architecture, is essential for appropriate interpretation. The anatomical location of the lesion in the mid-upper arm is also noteworthy. Although epitrochlear lymph node metastasis was considered, the lesion was situated more proximally than the typical epitrochlear nodal position, which is usually located a few centimeters above the medial epicondyle. This atypical location further complicated the distinction between interval nodal metastasis and ITM.

Taken together, these observations highlight that standardized classification and treatment algorithms for ITM in cSCC have not yet been established, reflecting the rarity and heterogeneity of this metastatic pattern [1,5-8]. From a practical standpoint, newly developed subcutaneous nodules along lymphatic drainage pathways after primary excision should prompt consideration of both ITM and interval nodal metastasis. Awareness of this pattern can help dermatologists and oncologic surgeons consider ITM in the differential diagnosis.

## Conclusions

In summary, this case illustrates a solitary lymphatic metastatic lesion arising along the lymphatic pathway from a dorsal forearm cSCC in an immunocompetent patient and underscores the importance of differentiating ITM from interval nodal metastasis in such presentations. The presence of residual lymphoid architecture within the lesion highlights the difficulty in distinguishing these entities and emphasizes the need for careful clinicopathological correlation. When a new subcutaneous nodule develops along the lymphatic drainage route after cSCC excision, both possibilities should be considered to ensure appropriate staging and management, as definitive classification may not always be possible in individual cases.
